# Identification of a new recombinant strain of echovirus 33 from children with hand, foot, and mouth disease complicated by meningitis in Yunnan, China

**DOI:** 10.1186/s12985-019-1164-2

**Published:** 2019-05-08

**Authors:** Jie Zhang, Hongbo Liu, Yilin Zhao, Haihao Zhang, Hao Sun, Xiaoqin Huang, Zhaoqing Yang, Jiansheng Liu, Shaohui Ma

**Affiliations:** 1 Institute of Medical Biology, Chinese Academy of Medical Sciences and Peking Union Medical College, Kunming, 650118 People’s Republic of China; 2 Yunnan Key Laboratory of Vaccine Research Development on Severe Infectious Disease, Kunming, 650118 People’s Republic of China

**Keywords:** HFMD, E-33, Recombination

## Abstract

**Background:**

Hand, foot, and mouth disease (HFMD) is a common childhood disease, which is usually caused by enterovirus A (EV-A) serotypes. Enterovirus A71 (EV-A71) and coxsackievirus A16 (CV-A16) are the main etiologic agents. Multiple serotypes of enterovirus B serotypes (EV-B) have been detected in outbreaks or sporadic cases of HFMD.

**Results:**

During HFMD surveillance in Yunnan, China in 2013, two echovirus 33 (E-33) isolates were recovered in cell culture and typed by molecular methods from the cerebrospinal fluid (CSF) and feces of two sporadic cases of HFMD complicated by meningitis. Sequence analysis indicated that the study isolates, YNK35 and YNA12, formed an independent branch, and belonged to E-33 genotype H. Recombination analysis indicated multiple recombination events in the genomic sequence of isolate YNK35. The recombination mainly occurred in the non-structural coding region of P2 and P3, and involved intra-species recombination of species B.

**Conclusion:**

In this study, the complete sequences of two E-33 isolates were determined. This is the first report of severe HFMD associated with E-33 in Yunnan China, and it enriches the number of full-length genome sequences of E-33 in the GenBank database.

**Electronic supplementary material:**

The online version of this article (10.1186/s12985-019-1164-2) contains supplementary material, which is available to authorized users.

## Background

Enteroviruses (EVs) are small, positive, single-stranded, non-enveloped RNA viruses belonging to the family *Picornaviridae*, which are further classified into fifteen species: enterovirus (EV)-A, EV-B, EV-C, EV-D, EV-E, EV-F, EV-G, EV-H, EV-I, EV-J, EV-K, and EV-L and rhinovirus (RV)-A, RV-B, and RV-C [1]. EVs cause a wide range of human diseases worldwide, including cutaneous, visceral, and neurological disorders. Among these diseases, hand, foot, and mouth disease (HFMD) is a common childhood disorder which typically presents as a brief, febrile illness characterized by the association of oral ulcerations (enanthema) and vesicular rash (exanthema) on the palms, soles, and buttocks [2]. EV-A71 and CV-A16 are the major etiological agents of HFMD [3, 4]. Serotypes of EV-B (Es) have been mainly related to herpangina and seasonal outbreaks of mild viral meningitis, but have recently been associated with epidemics of HFMD. Echovirus (E) belongs to species EV-B. And multiple E serotypes are detected in sporadic cases or outbreaks of HFMD, and frequently co-circulated with EV-A71 and CV-A16 in large epidemics, including E-3, E-4, E-5, E-6, E-7, E-9, E-11, E-16, E-17, E-18, E-24, E-25, and E-30 [5–22].

In 1959, echovirus 33 (E-33) was isolated, and was identified as the last Enteric Cytopathic Human Orphan (ECHO) virus belonging to the species enterovirus B in 1963 [23]. Since then, E-33 has been detected in several outbreaks and sporadic cases around the world, associated with rashes, diarrhea, fever, aseptic meningitis, encephalitis, acute flaccid paralysis (AFP), and influenza-like illness [24–29]. The prototype strain (Toluca-3) of E-33 was isolated in 1959 [23]. To date, there are only two full-length genome sequences in the GenBank database, including the prototype strain and another E-33 strain isolated in 2014, and clinical information on E-33 has not been reported. During HFMD surveillance, two E-33 viruses were isolated, from stool and CSF, and identified in two sporadic cases of HFMD complicated by meningitis. To our knowledge, this was the first study to report severe HFMD caused by E-33 in Yunnan China.

## Results

### Primary characterization of the virus isolates

The isolates YNK35/CHN/2013 and YNA12/CHN/2013 were recovered from human embryonic lung diploid fibroblast (KMB17), human rhabdomyosarcoma (RD), and human lung cancer (A549) cell lines. YNK35 and YNA12 were isolated from CSF and stool samples from two sporadic cases of severe HFMD complicated by meningitis. Capsid protein VP1 sequencing and molecular typing using BLAST [30] revealed that these two study isolates were E-33.

When compared with the entire VP1 coding sequences of different prototype EVs retrieved from GenBank, YNK35 and YNA12 were found to have high homology with E-33, with 78.2–78.3% identity to the E-33 prototype strain, Toluca-3, but < 70% identity to the VP1 sequences of all other enterovirus serotypes. It is proposed that YNK35 and YNA12 isolates belong to E-33, according to the enterovirus type demarcation criterion (that strains with > 75% nucleotide or > 88% amino acid homology of VP1 sequences belong to the same serotype).

### VP1 sequence analysis

**Fig. 1:**
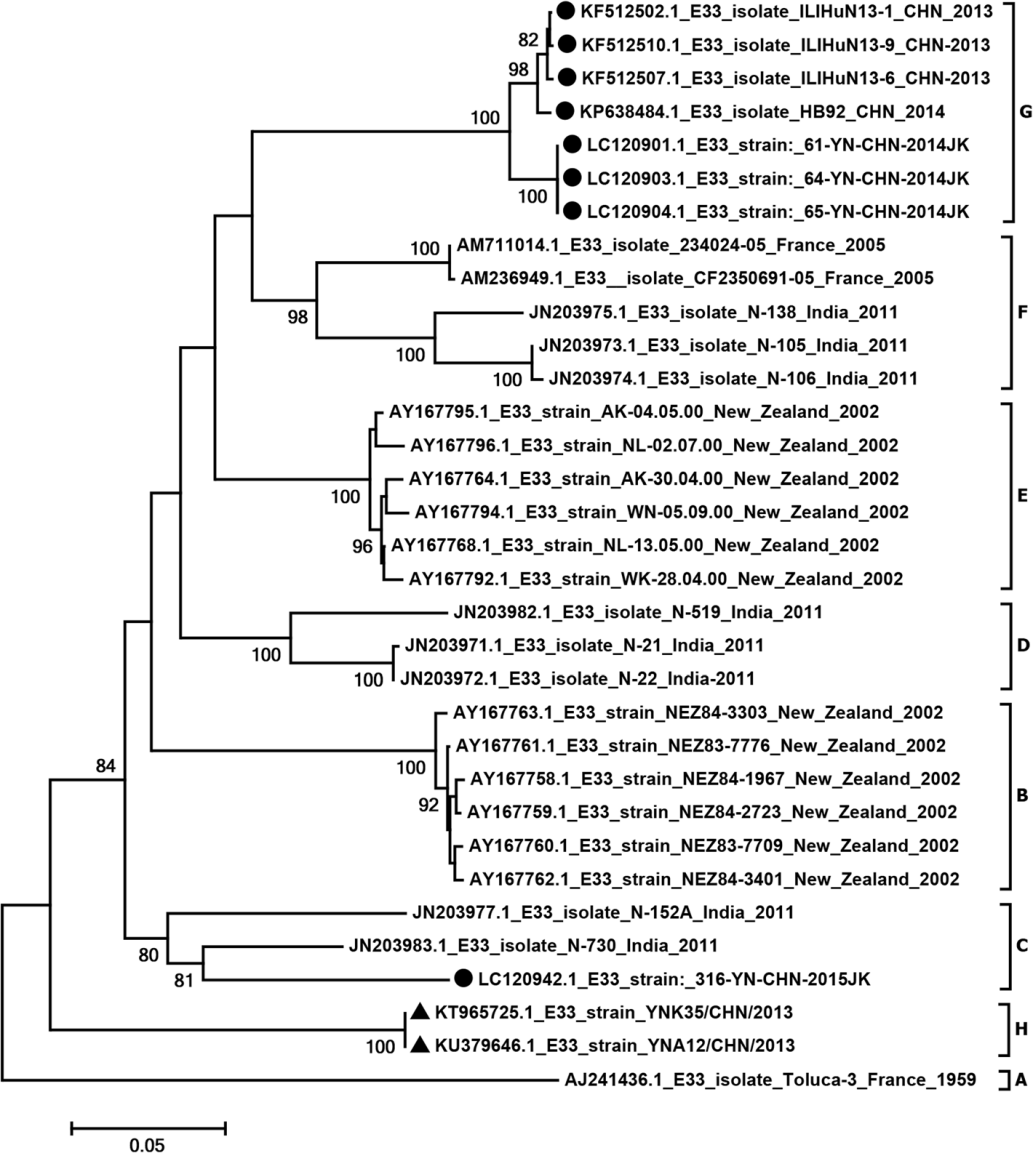
Phylogeny of the complete *VP1* sequences of 98 E-33 strains. The MEGA 6.06 program was utilized to analyze the relationships between the two Yunnan E-33 isolates and the other 96 E-33 isolates available in the GenBank database. Numbers at the nodes represent bootstrap values supported for that node (1000 bootstrap replicates). Only high bootstrap values (>75%) are shown. The scale bars indicate the genetic distance. ▲ strains were isolated in this study, and ● strains were collected from other provinces in China. The Complete trees are available as Additional file 1: Figure S1

Phylogenetic trees were constructed by aligning the complete VP1 sequences of the two E-33 study isolates with other E-33 sequences retrieved from GenBank. In order to study the genetic diversity and possible relationships of the E-33 strains, 31 E-33 sequences available in GenBank were collected from different country or different date. The phylogenetic tree revealed that global E-33 strains could be grouped into eight major distinct phylogenetic clusters, corresponding to A–H (Fig. 1). Prototype Toluca-3 was the only strain in cluster A. The topology of the phylogenetic tree indicated that the study Yunnan isolates YNK35 and YNA12 isolated in 2013 belonged to genotype H and formed an independent branch. However, one Yunnan isolate (316-YN-CHN-2015JK) isolated in 2015 belonged to genotype C. Most Chinese E-33 isolates, ILIHuN13–1, ILIHuN13–6, ILIHuN13–9, 61-YN-CHN-2014JK, 64-YN-CHN-2014JK, 65-YN-CHN-2014JK, and HB92 clustered as genotype G.

Homology comparisons of the complete *VP1* nucleotide and amino acid sequences were performed among the E-33 genotypes (Additional file 1: Table S1). The average *VP1* nucleotide sequence divergence within the E-33 genotypes was 17.5% (12.1–22.9%). In particular, for genotype H, which had nucleotide divergence of 17.75% (17.1–18.4%) and 17.0% (16.3–17.7%), respectively. When compared with genotypes G and C, all sequences were above the mean 14.95% cutoff divergence value assigned for EV-A71 subgenotyping [31]. YNK35 and YNA12 had 81.6–82.6% (98.6–99.3%) nucleotide (amino acid) sequence similarity with other Chinese E-33 isolates with complete *VP1* sequences.

### Complete genome analysis

Isolates YNK35 and YNA12 were found to be closely related, having 99.0% nucleotide (98.2% amino acid) identity for the entire genome, so the complete genomic sequence of isolate YNK35 was characterized as representative in the following analysis. The complete genome of isolate YNK35 consisted of 7392 nucleotides, including a 6552-nucleotide open reading frame (ORF) encoding a potential polyprotein precursor of 2183 amino acids, a 5′-untranslated region (UTR) of 743 nucleotides and a 3′-UTR of 97 nucleotides. In comparison with the prototype strain Toluca-3, it had 79.9% similarity and 95.7% similarity in the complete genome and deduced amino acid sequences of the potential polyprotein precursor, respectively. No nucleotide deletions or insertions were found in alignment of isolate YNK35 and the prototype strain. In addition, isolate YNK35 had 81% nucleotide and 96.4% amino acid similarity, respectively, with the only Chinese E-33 isolate with a complete genome deposited in the GenBank, which was named HB92 but was not typed as E-33 by the authors. Table 1 shows a comprehensive comparison of different genomic regions of the nucleotide sequence and the deduced amino acid sequence of isolate YNK35 with the E-33 prototype strain and HB92 isolate.

**Table 1:** Nucleotide and amino acid identities between Toluca-3/HB92 and YNK35/CHN/2013 in different genomic regions

Genomic region	Toluca-3		HB92	
	% Nucleotide identity	% Amino acid identity	% Nucleotide identity	% Amino acid identity
5’UTR	83.4		85.3	
VP4	79.0	97.1	79.2	98.6
VP2	77.9	90.8	81.0	94.9
VP3	78.4	95.4	81.8	97.1
VP1	78.2	96.4	82.4	99.3
2A	77.1	92.0	81.1	94.7
2B	76.1	95.9	78.2	93.8
2C	81.0	99.4	80.2	98.2
3A	81.3	93.3	80.9	95.5
3B	77.0	95.0	80.3	100
3C	80.9	98.9	77.4	97.3
3D	81.3	96.3	80.7	96.3
3’UTR	84.4		84.5	
Complete genome	79.9	95.7	81.0	96.4

### Phylogenetic analysis based on *P1*, *P2*, *P3* coding regions

**Fig. 2:**
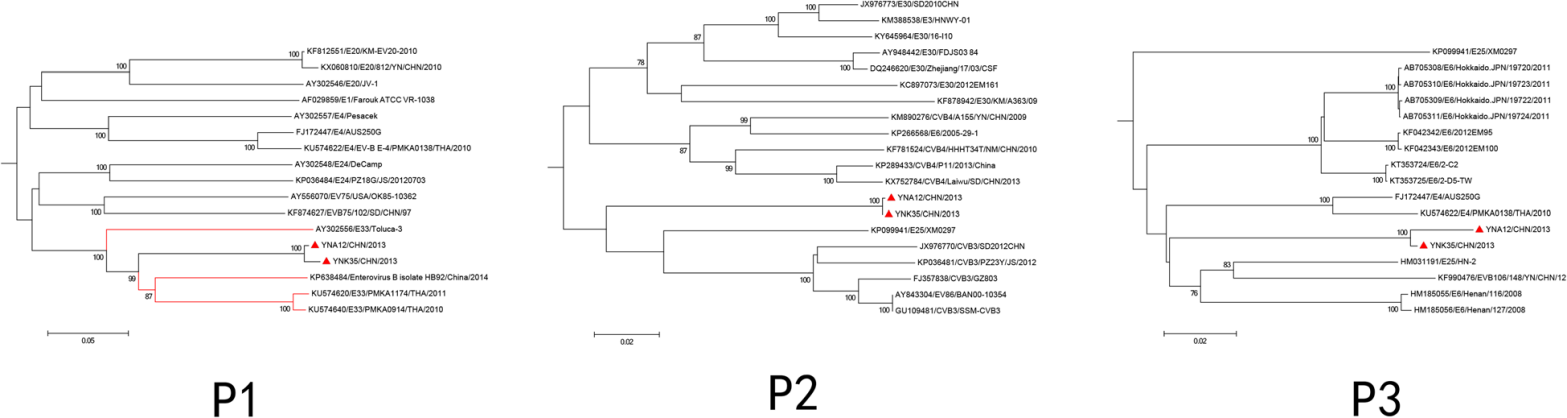
Phylogenetic trees based on the *P1*, *P2*, and *P3* coding sequences of 383 EV-B strains. The phylogenetic relationships among the two E-33 isolates and 381 EV-B strains available in the GenBank database were analyzed using the MEGA 6.06 program. Numbers at the nodes represent bootstrap values supported for that node (1000 bootstrap replicates). Only high bootstrap values (>75%) are shown. The scale bars indicate the genetic distance. ▲ strains were isolated in this study and the red colour indicates the other E-33 strains

Construction of phylogenetic trees by aligning the sequences of the coding regions *P1*, *P2*, and *P3* of YNK35 and YNA12 with 383 EV-B strains available in the GenBank database are shown in Fig. 2. In the *P1* capsid coding region, YNK35 and YNA12 formed a lineage, with a bootstrap value of 100%. Meanwhile, the two study isolates formed a cluster with all other E-33 strains, such as Toluca-3, HB92 and PMKA0914/1174 (which were isolated from undiagnosed respiratory specimens in Thailand [32]), correspondent with the preliminary molecular typing results based on the complete VP1 sequences.

The phylogeny of the non-structural protein coding regions displayed different results; the isolates YNK35 and YNA12 were interspersed with other EV isolates. In the *P2* coding region, YNK35 and YNA12 were closely related to CV-B3, EV-86, and E-25. In the *P3* coding region, YNK35 and YNA12 were very similar to E-4, EV-B106, and E-6. These results suggested that one or more putative recombination events had probably occurred between the study isolates and other EV serotypes.

### Recombination analysis

**Fig. 3:**
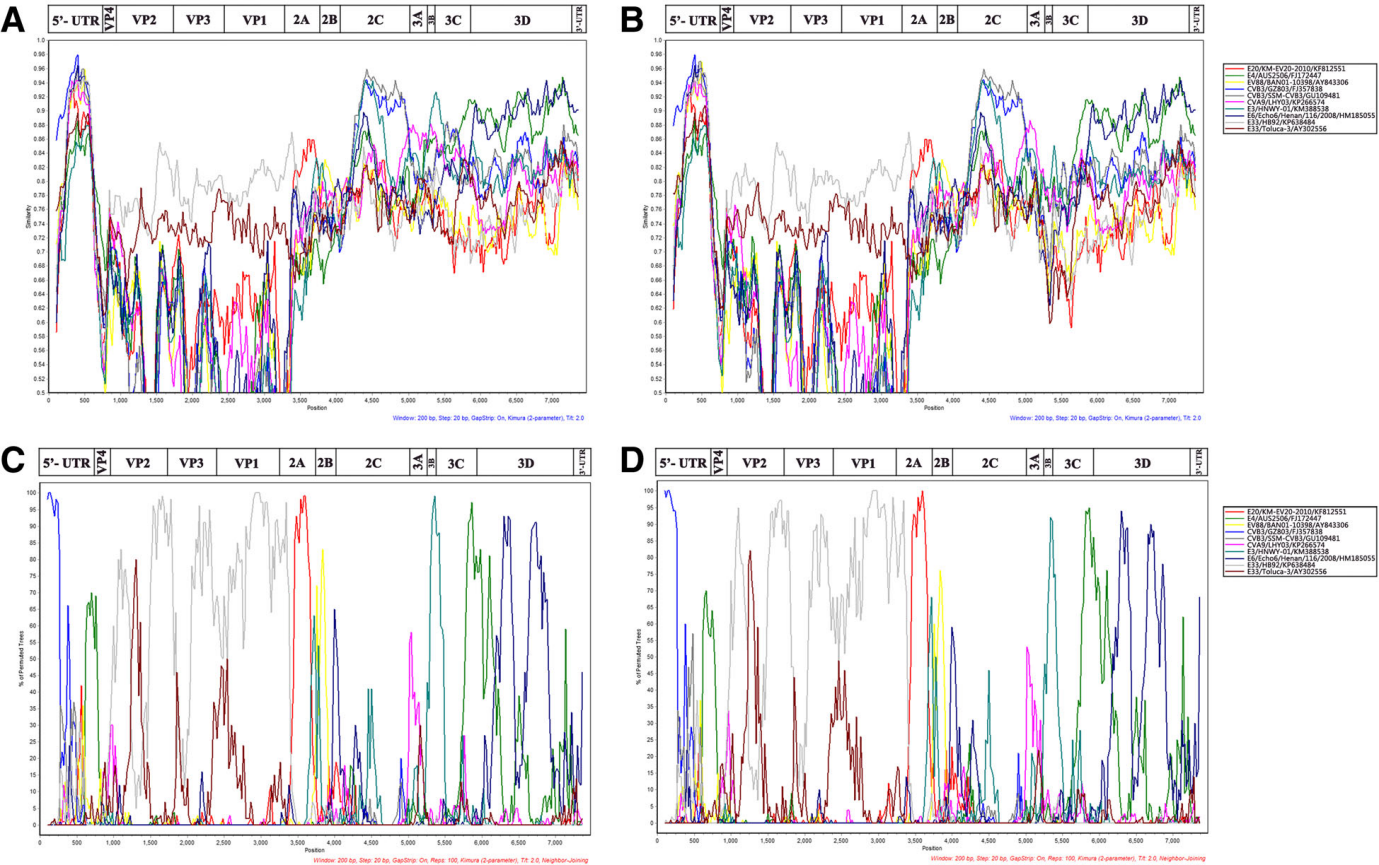
Similarity plot and bootscanning analyses of the complete genome of the two Yunnan E-33 strains. Similarity plot (**a**/**b**) and bootscanning analysis (**c**/**d**) of complete EV-B genomes use a sliding window of 200 nucleotides moving in steps of 20 nucleotides. The genome of isolate YNK35/CHN/2013 (**a**/**c**), YNA12/CHN/2013 (**b**/**d**) serves as a query sequence, independently

To confirm the recombination events occurring between the Yunnan E-33 isolates and other EV-B serotypes, a similarity plot was produced and bootscanning analyses were performed (Fig. 3). The similarity plot and bootscanning analyses both suggested multiple recombination events in the genomic sequence of isolates YNK35 and YNA12. In the *P1* and *2C* coding regions, isolate YNK35 had the highest identity with the E-33 isolate HB92. However, in the 5′-UTR, isolate YNK35 was most homologous with the CV-B3 strain GZ803. For the *2A* coding region, it was most similar to the E-20 strain KM-EV20–2010. In the *3B* coding region, YNK35 showed highest identity with the E-3 strain HNWY-01. In addition, YNK35 showed highest identity with the E-4 strain AUS250G and E-6 strain Echo6/Henan/116/2008, in the partial *3C* coding region and the *3D* coding region, respectively.

## Discussion

In this study, YNK35 was isolated from CSF, and YNA12 was isolated feces. The patients displayed a similar clinical presentation: hand, foot, and mouth disease complicated by aseptic meningitis. Both isolates produced typical enterovirus-like cytopathic effect (CPE) on KMB17, RD, and A549 cell lines; enterovirus molecular serotyping confirmed the presence of E-33. The E-33 isolate YNK35 was isolated from CSF, which is the best specimen for the conclusive identification of the pathogenic agent in a patient with meningitis. The Chinese E-33 isolates, ILIHuN13–1, ILIHuN13–6, and ILIHuN13–9, were responsible for a large outbreak of influenza-like illness (ILI) in Hunan Province, China in 2013 [25]. Other isolates, 61-YN-CHN-2014JK, 64-YN-CHN-2014JK, 65-YN-CHN-2014JK and 316-YN-CHN-2014JK were detected in healthy children. And no clinical information, molecular epidemiology, or disease correlation of the Chinese isolate HB92 has been reported. Therefore, the etiological characteristics of E-33 in China still need further study.

Sequence analysis indicated that the study isolates YNK35 and YNA12 could be regarded as a separate genotype in the near future, whereas the contemporary Chinese E-33 isolates, ILIHuN13 and HB92 were clustered as genotype G. This implies that there were at least two E-33 genotypes circulating in mainland China in that era. In addition, previous research confirmed that partial sequences of sporadic E-33 strains were identified in feces from healthy children in Yunnan, China [33]. Therefore, before these pathogenic E-33 strains appeared, they may have formed a new genomic lineage that had been circulating “silently” for some years within the same geographical area.

Recombination drives the evolution of EVs and usually occurs in the non-structural regions of EV-B [34–38]. The recombination analysis suggested that multiple recombination events had occurred in the genomic sequence of isolates YNK35 and YNA12. In this study, recombination events were detected between isolate YNK35 (or YNA12) and other EV-B strains, such as EV-86 strains, CV-B3 strains, E-4 strains, and the EV-B106 strain. The recombination had mainly occurred in the non-structural coding region of *P2* and *P*3 among EV-B strains, representing the intra-species recombination. The recombination donors were usually contemporary predominant circulating EV-B; it is worth noting that they were isolated from different geographical locations around the world. Whether or how these recombinations may have contributed to the emergence of lineages with modified pathogenic properties (such as severe HFMD) also needs to be investigated further.

## Conclusion

In summary, we have reported the entire genome sequences of two E-33 isolates isolated during HFMD surveillance in Yunnan, China in 2013. Analysis of the nucleotide sequences revealed that these two Yunnan isolates have high genetic diversity when compared with the E-33 prototype strain, showing intertypic recombination in the non-structural protein encoding region, and genetic exchanges with other EV-B strains. Until now, only two E-33 isolates with complete genome sequences have been deposited in GenBank. The study enriched the number of full-length genome sequences of E-33 in the GenBank database, as well as contributing to the molecular epidemiology of E-33 in China, which is of great importance when evaluating the association between E-33 and E-33-related diseases.

## Methods

### Samples and virus isolation

During HFMD surveillance (China Information System for Disease Control and Prevention) in 2013, two patients with HFMD (with rashes on hands, feet, knees, and buttocks), a 1-year and 6-month-old girl and a 1-year and 2-month-old boy, were admitted to a pediatric hospital in Kunming (China). Both children manifested nonspecific fever of 38.9–39.3 °C and meningeal signs of a sore or stiff neck. CSF and stool specimens were collected from the two patients. To make a thorough attempt to isolate poliovirus, RD, A549, and KMB17 cell lines were used, in accordance with standard protocol [39]. All positive isolates (cell cultures with CPE appearance) were stored at − 80 °C until use.

### Viral RNA extraction, reverse transcription-polymerase chain reaction (RT-PCR), sequencing and typing

Nucleic acid was extracted from cell culture supernatants with an AxyPrep Body Fluid Viral DNA/RNA Miniprep Kit (Axygen, Union City, CA, USA), which was used in accordance with the manufacturer’s instructions. The RT-PCR reactions were performed with a PrimeScript™ One Step RT-PCR Kit Ver.2 (Takara, Dalian, China) to amplify the partial VP1 gene by using VP1 gene-specific primer pairs 222 and 224 [40]. These sequences of partial VP1 were uploaded into GenBank BLAST (http://www.ncbi.nlm.nih.gov/BLAST/) for direct identification of enterovirus serotypes. The primers (Additional file 1: Table S2) used for amplification of the complete genomes were based on the prototype strain, and were designed using a “primer-walking” strategy [41]. An ABI 3730XL automatic sequencer (Applied Biosystems, Foster City, CA, USA) was used to sequence the resulting DNA templates at Tsingke Biological Technology Co., Ltd., Beijing.

### Sequence analysis and recombination analysis

Phylogenetic trees were constructed by Molecular Evolutionary Genetic Analysis (MEGA) version 6.06 software, using the neighbour-joining algorithms with 1000 bootstrap replicates. Simplot software version 3.5.1 was used to analyze the plot of nucleotide similarities, with a sliding window of 200 nucleotides moving in 20-nucleotide steps [41]. Pairwise alignments of the sequences were carried out via Geneious Basic 5.6.5 software [41].

### The GenBank accession number of the nucleotide sequences

The complete genome sequences of the E-33 isolates YNK35/CHN/2013 and YNA12/CHN/2013 identified in this study are available under the GenBank accession numbers KT965725 and KU379646.

## Additional file

Additional file 1: Supplementary Information. (DOC 63250 kb)

## Abbreviations

5′UTR: 5′-untranslated region
CPE: cytopathic effect
E-33: Echovirus 33
Es: Echoviruses
EVs: Enteroviruses
