# Supplementary material for: Identification of a new recombinant strain of echovirus 33 from children with hand, foot, and mouth disease complicated by meningitis in Yunnan, China
Source: Virol J. 2019 May 8;16:63. doi: 10.1186/s12985-019-1164-2 (PMC6506940; doi:10.1186/s12985-019-1164-2)
Supplement: Supplementary file 1 — Supplementary Information. (DOC 63250 kb) [file 12985_2019_1164_MOESM1_ESM.doc]

Supplementary Information

**Identification of a new recombinant strain of Echovirus 33 from children with hand, foot, and mouth disease complicated by meningitis in Yunnan, China**

**Jie Zhang****^1,2^*****, Hongbo Liu^1,2^*****,Yilin Zhao^1,2^,** **Haihao Zhang** **^1,2^, Hao Sun^1,2^,** **Xiaoqin Huang ^1,2^,Zhaoqing Yang^1,2^, Jiansheng Liu^1,2^🖂＆,Shaohui Ma****^1,2^🖂**

**^1^** Institute of Medical Biology, Chinese Academy of Medical Sciences and Peking Union Medical College, Kunming 650118, PR China. ^2^ Yunnan Key Laboratory of Vaccine Research Development on Severe Infectious Disease, Kunming 650118, PR China.

*Jie Zhang and Hongbo Liu have contributed equally to this study. Correspondence and requests for materials should be addressed to J.L. (email: ljsh3300@163.com) or S.M. (email: [shaohuima70@hotmail.com](mailto:shaohuima70@hotmail.com))

***Supplementary Figure S1*. Complete Phylogenetic trees based on the *P1*, *P2*, and *P3* coding sequences of 383 EV-B strains.** The phylogenetic relationships among the two E-33 isolates and 381 EV-B strains available in the GenBank database were analyzed using the MEGA 6.06 program. Numbers at the nodes represent bootstrap values supported for that node (1,000 bootstrap replicates). Only high bootstrap values (>75%) are shown. The scale bars indicate the genetic distance. ▲ strains were isolated in this study and the red colour indicates the other E-33 strains.

***Supplementary Table S1*. The VP1 nucleotide and amino acid homology between the E-33 gene clusters**

| cluster | A | B | C | D | E | F | G | H |
| --- | --- | --- | --- | --- | --- | --- | --- | --- |
| A |  | 77.1-77.3 | 77.8-79.0 | 77.3-77.7 | 77.5-82.9 | 76.6-78.3 | 76.5-76.6 | 78.2 |
| B | 96.4-96.8 |  | 84.6-85.3 | 84.9-86.2 | 86.0-87.6 | 86.5-89.2 | 83.5-84.1 | 82.6-82.9 |
| C | 96.1-96.4 | 97.5-98.2 |  | 86.0-88.4 | 83.3-83.6 | 82.4-82.6 | 83.5-86.2 | 82.3-83.7 |
| D | 96.1-97.2 | 96.8-98.2 | 96.4-97.9 |  | 86.7-89.2 | 83.4-86.2 | 84.8-86.2 | 83.2-83.3 |
| E | 94.7-97.5 | 96.8-98.2 | 96.8-97.9 | 96.4-99.6 |  | 87.3-89.8 | 85.8-87.9 | 83.3-83.6 |
| F | 96.4-96.8 | 97.2-98.2 | 96.8-98.6 | 96.8-100 | 96.8-99.6 |  | 87.0-87.1 | 82.4-82.6 |
| G | 95.7-96.4 | 97.9-98.6 | 97.5-98.2 | 97.5-99.3 | 97.5-98.9 | 98.6-99.6 |  | 81.6-82.9 |
| H | 95.0-97.2 | 97.9-98.6 | 98.2-98.9 | 98.2-98.9 | 97.9-98.9 | 96.8-98.6 | 98.6-99.3 |  |

Note: The data in the lower left corner represent the amino acid homology comparisons, and those in the upper right corner represent the nucleotide homology comparisons.

***Supplementary Table S2*.** **Primers used for amplifying and sequencing the complete genomes**

| Name | Sequence (5′→ 3′) | Position | **Orientation** |
| --- | --- | --- | --- |
| 224 | CICCIGGIGGIAYRWACAT | 2969-2951 | Forward |
| 222 | GCIATGYTIGGIACICAYRT | 1977-1996 | Reverse |
| EV1F | TTAAAACAGCCTGTGGGTTG | 1-20 | Forward |
| E332R | TATAAATCCAGCATCAGTGT | - | Reverse |
| E333F | AAGTTGCCCAGCTTCGCAG | - | Forward |
| E335R | TGCAAACATATGAAGGCC | 5220-5203 | Reverse |
| E335F1 | GGACATGTTGGTCACCGAG | - | Forward |
| EV8R | CACCGAATGCGGAGAATTTA | 7392-7373 | Forward |
| E331R | TTCTCCTTCAACCGCGT | - | Reverse |
| E332F | ACTTTGTGTTATATTGTC | - | Forward |
| E334F | GCTTTGAGGGACCAGGCCT | - | Forward |
| E336F | TTGAATGATTCTGTTGCG | - | Forward |
| E335F2 | CCTCATGAATGAGCAAGA | - | Forward |

Note: F (forward) and R (reverse) indicate the orientation of each primer; -: indetermin
